# Supplementary material for: The human health burden of non-typhoidal Salmonella enterica and Vibrio parahaemolyticus foodborne gastroenteritis in Shanghai, east China
Source: PLoS One. 2020 Nov 13;15(11):e0242156. doi: 10.1371/journal.pone.0242156 (PMC7665802; doi:10.1371/journal.pone.0242156)
Supplement: S1 Questionnaire — (DOC) [file pone.0242156.s001.doc]

**Study Questionnaire**

Date of interview Years/Months/Days

**Section one: Basic information**

1. Sentinel site code

2. Person code

3. If the questionnaire being answered by the selected respondent him or herself?

[1] Yes [2] No

4. How many people are usually live in this household?

4a. How many are <18 years of age?

5. Total family income per year yuan

6. Home address

Province Prefecture District/County

Township/Street Village/Community

7. Residence [1] Urban [2] Rural

8. Telephone

9. What is your (your child’s) name?

10. Gender [1] Male [2] Female

11. Age years

12. Ethnic group [1] Han [2] Manchu [3] Mongol [4] Hui [5] Tibetan [6] Miao

[7] Zhuang [8] Uyghur [9] Other

13. Education

[1] Preschool children [2] Illiterate [3] Primary school [4] Secondary school

[5] High school [6] Technical secondary school and junior college

[7] University [8] Postgraduate

14. Occupation

[1] Teacher [2] Worker [3] Migrant labourer [4] Peasant [5] Herdsman

[6] Fisherman [7] Housekeeper [8] Employed persons in catering services

[9] Business service personnel [10] Administrator/Director [11] Office staff

[12] Research specialist staff [13] medical personnel [14] Self-employed

[15] Retired [16] Unemployed [17] Too young to work (including students)

[18] Other

15. During the past two weeks did you (your child) travel outside of the district/county where your household resides? [1] Yes [2] No

**Section two: Symptoms**

Acute gastrointestinal illness was defined as diarrhoea of ≥ 3 loose stools in a 24-hour period or significant vomiting with at least one other symptom (abdominal pain/cramps, fever), but excluding those (a) with Crohn’s disease, irritable bowel syndrome, colitis, diverticulitis of large intestine, or another chronic illness with symptoms of diarrhoea or vomiting, or (b) who report their symptoms were due to pregnancy, excess alcohol, chemotherapy/radiotherapy, drugs, or food allergy.

16. During the past 4 weeks, have you (your child) suffered from diarrhoea?

[1] Yes [2] No

16a. If yes, how many times per day?

17. Have you (your child) suffered from bloody diarrhoea? [1]Yes [2] No

17a. If yes, how much blood was there in your (your child’s) stool?

[1] Just a little blood on the toilet paper

[2] Some blood mixed with the stool

[3] So much blood that the stool was almost entirely blood

18. Have you (your child) suffered from vomiting? [1] Yes [2] No

18a. If yes, how many times per day?

If you have vomiting, did you (your child) also experience any of the following symptoms?

18b. Diarrhoea [1] Yes [2] No

18c. Abdominal pain/cramps [1] Yes [2] No

18d. Fever [1] Yes [2] No

19. Are you (your child) still suffering from any of the above symptoms?

[1] Yes [2] No

20. How long did the diarrhoea last? Days Hours

21. Cause of the illness

21a. What do you think caused your (your child’s) symptoms?

[1] Food poisoning [2] Person-to-person [3] Contaminated water

[4] Animal contact [5] Other [6] Unknown

21b. Other, please specify

21c. Do you think that your (your child’s) symptoms are caused by the following condition, such as Crohn’s disease, irritable bowel syndrome, colitis, 0000000000000000000000000000000000000000000000000000000000000000000000000000000000000000000000000000000000000000000000000 9]以下情况 00000000000000000000000000000000000000000000000000000000000000000000000000000000000000000000000000000000000000000000000diverticulitis of large intestine, pregnancy, excess alcohol, chemotherapy/radiotherapy, medication, food allergy? [1] Yes [2] No

22. A 7-day symptom-free interval was defined to distinguish multiple episodes. Judged by the interviewer, how many episodes of acute gastrointestinal illness did the respondent have during the past 4 weeks?

**Section three: Suspected food**

23. If food poisoning, which food you think was most suspected to cause your (your child’s) symptoms?

23a. Type of food

[1] Meat and meat products [2] Milk and dairy products

[3] Eggs and egg products [4] Fishery products [5] Cereals and cereal products

[6] Beans and bean products [7] Vegetable Oil [8] Fruits and vegetables

[9] Other [10] Unknown

24. If food poisoning, where do you think you (your child) got the food that caused your (your child’s) symptoms?

[1] Own home [2] Private house (excluding own home) [3] Hotel/Restaurant

[4] Fast food service [5] Food supermarket [6] Street vender [7] Takeaway

[8] School cafeteria [9] Company cafeteria [10] Food service on construction sites

[11] Other [12] Unknown

24a. Other, please specify

**Section four: Medical treatment**

25. As a result of this illness for how many times did you (your child) visit a doctor?

(enter ‘0’ if none)

26. If visited a doctor, did you visit a doctor because you wanted diagnosis and treatment or required certificate for work? [1] Yes [2] No

26a. Wanted diagnosis and treatment [1] Yes [2] No

26b. Required certificate for work [1] Yes [2] No

27. As a result of this illness for how many days were you (your child) hospitalized?

(enter ‘0’ if none)

28. Were you (your child) asked to submit a stool sample? [1] Yes [2] No

28a. The result of the stool sample (enter the etiology being identified by the laboratory, if not sure, enter ‘unknown’)

29. Did you (your child) take any medications for this illness? [1] Yes [2] No

29a. Pharmacy [1] Yes [2] No

29b. Hospitals with prescription [1] Yes [2] No

29c. Family medicine chest [1] Yes [2] No

29d. Other, please specify

30. How many days were medications taken for?

31. Name of the medication(s) (enter ‘unknown’ if not sure)

Type of medicine

31a. Antibiotics [1] Yes [2] No

31b. Antidiarrhoeals [1] Yes [2] No

31c. Analgesics [1] Yes [2] No

31d. Antipyretics [1] Yes [2] No

31e. Antacids [1] Yes [2] No

31f. Other [1] Yes [2] No

31g. Unknown [1] Yes [2] No

**Section five: Social and economic impact of illness**

32. Did this illness require you (your child) to miss work or school/college?

[1] Yes [2] No

32a. Days missed from work (enter ‘0’ if none)

32b. Days missed from school/college (enter ‘0’ if none)

33. Did anyone else in your household have similar symptoms? If yes, how many?

(enter ‘0’ if none)

Interviewer Assessor

调查表

调查日期： 年 月 日

一、基本情况

1监测地区编码

2个体编码

3是否本人接受调查？[1]是 [2]否

4家庭常住 人

4a 其中＜18岁 人

5家庭年收入 元

6家庭住址： 省/自治区/直辖市 （地级）市 市/县/区

乡镇/街道 村/（社区）居委会

7居住地性质：[1]城市 [2]农村

8联系电话：

9（家长）姓名：

10性别：[1]男 [2]女

11年龄 岁

12民族：[1]汉族 [2]满族 [3]蒙古族 [4]回族 [5]藏族 [6]苗族

[7]壮族 [8]维吾尔族 [9]其他

13文化程度：[1]未到入学年龄 [2]文盲 [3]小学 [4]初中 [5]高中

[6]大中专 [7]大学 [8]研究生

14职业：[1]教师 [2]工人 [3]民工 [4]农民 [5]牧民 [6]渔民 [7]保姆

[8]餐饮业从业人员 [9]商业服务人员 [10]干部 [11]职员

[12]科研人员 [13]医疗人员 [14]个体 [15]离退人员 [16]无业人员

[17]未到就业年龄（包括学生） [18]其他

15过去2周是否因为出差旅游等离开所居住的区（县）？[1]是 [2]否

二、症状

急性胃肠炎是指每日排便3次或以上或呕吐，如果为呕吐，要求同时伴有腹痛、发热中的一种或一种以上。克罗恩病、肠易激综合征、结肠炎、大肠憩室炎、妊娠、过量饮酒、化疗/放疗、药物治疗、食物过敏等不属于引起急性胃肠炎的原因。

16过去4周，您（您的孩子）是否出现腹泻？[1]是 [2]否

16a 如出现腹泻，24小时内腹泻 次

17是否出现血性腹泻？[1]是 [2]否

17a如出现血性腹泻，粪便的血量为

[1]少量，卫生纸上看到血

[2]中等量，粪便中混合着血

[3]大量，血非常得多，粪便几乎是红色的

18是否出现呕吐？[1]是 [2]否

18a 如出现呕吐，24小时内呕吐 次

如果出现呕吐，那么是否还出现以下症状？

18b腹泻？[1]是 [2]否

18c腹痛？[1]是 [2]否

18d发热？[1]是 [2]否

19是否您（您的孩子）今天仍有上述症状？[1]是 [2]否

20腹泻持续时间 天 小时

21您（您的孩子）是因为以下什么原因导致出现上述急性胃肠炎症状?

21a 是因为以下原因？

[1]食物中毒 [2]人与人的传染 [3]水 [4]动物接触 [5]其他 [6]不明

21b如果为其他原因，请具体说明

21c 是因为下列原因？[1]是 [2]否

[1]克罗恩病 [2]肠易激综合征 [3]结肠炎 [4]大肠憩室炎 [5]妊娠

[6]过量饮酒 [7]化疗/放疗[8]药物治疗 [9]食物过敏

22在过去的28天中，间隔7天再次出现急性胃肠炎，可以填写为2次急性胃肠炎。调查人员综合判断，过去4周调查对象共发生了 次急性胃肠炎

三、可疑饮食史

23如果怀疑是食物中毒，您认为最可疑的食物是

23a其所属食物类别为：

[1]肉与肉制品 [2]乳与乳制品 [3]蛋与蛋制品 [4]水产品 [5]谷类及制品

[6]豆类及制品 [7]植物油 [8]果蔬类 [9] 其他 [10]不明

24如果怀疑是食物中毒，您认为您（您的孩子）是在哪里获得引起症状的食物？

[1]家中 [2]他人的家中 [3]宾馆饭店 [4]快餐店 [5]食品超市

[6]街头摊点 [7]送餐 [8]学校食堂 [9]单位食堂 [10]工地食堂

[11]其他 [12]不明

24a如果为其他地点，请具体说明

四、治疗情况

25您（您的孩子）因病去医院就诊 次（没有则填0）

26如果去医院就诊，是否是因为要得到诊断和治疗或因为要获得休假证明？

[1]是 [2]否

26a获得诊断和治疗：[1]是 [2]否

26b获得休假证明： [1]是 [2]否

27您（您的孩子）因病住院 天（没有则填0）

28您（您的孩子）是否被要求提供粪便样本？[1]是 [2] 否

28a实验室检测结果 （填检测出何致病因子，若不清楚则填不详）

29您（您的孩子）是否因病吃药？[1]是 [2]否

29a药店药物？ [1]是 [2]否

29b根据医生处方在医院拿药？ [1]是 [2]否

29c家庭自备药物？ [1]是 [2]否

29d其他，请具体说明

30服用药物 天

31药物名称 （若不清楚则填不详）

药物所属类别

31a止泻药? [1]是 [2]否

31b抗生素？[1]是 [2]否

31c止痛药？[1]是 [2]否

31d退热剂？[1]是 [2]否

31e解酸药？[1]是 [2]否

31f其他？ [1]是 [2]否

31g不明？ [1]是 [2]否

五、疾病的社会经济影响

32您（您的孩子）是否因病旷工或旷课？[1]是 [2]否

32a旷工 天（没有则填0）

32b旷课 天（没有则填0）

33家中其他成员 人有类似的症状（没有则填0）

调查员签字： 审核员签字：
